# Parturition in baboons (*PAPIO SPP*.)

**DOI:** 10.1038/s41598-018-19221-4

**Published:** 2018-01-19

**Authors:** N. Schlabritz-Loutsevitch, J. Maher, R. Sullivan, G. Mari, M. Schenone, H. L. Cohen, R. A. Word, G. B. Hubbard, E. J. Dick

**Affiliations:** 1 Department of Obstetrics and Gynecology, College of Medicine, Texas Tech University Health Sciences Center at the Permian Basin, Odessa, TX USA; 2 Department of Comparative Medicine, University of Tennessee Health Science Center, Memphis, TN USA; 3 Department of Obstetrics and Gynecology, University of Tennessee Health Science Center, Memphis, TN USA; 4 Department of Radiology, University of Tennessee Health Science Center, Memphis, TN USA; 5 University of Texas Southwestern Medical Center, Dallas, TX USA; 6 University of Texas Health Sciences Center at San Antonio, San Antonio, TX USA; 7 Southwest National Primate Research Center, Texas Biomedical Research Institute, San Antonio, TX USA

## Abstract

The Old World non-human primates (NHP) - baboons (*Papio spp*.) share similarities with humans regarding fetal and placental development and some pregnancy-related complications. Information about the mechanism of birth and complications arising during parturition in these species is relatively sparse. In this manuscript, we add information from a series of pathological and observational cases to highlight insights and selected complications of birth in *Papio spp*, based on video-recording of the delivery process, X-ray, MRI, and ultrasound evaluations in pregnant baboons. Additionally, we abstracted pathology records obtained from perinatal loss in a large baboon colony during a 17 year period. The presented cases provide important information for the management of pregnancy and delivery in *Papio spp*.

## Introduction

Successful vaginal delivery involves an intricate interaction between the fetus and the maternal bony pelvis as the infant negotiates the birth canal via a mechanism of rotations of the presented part during the descent through the maternal pelvis. Increased fatness of the infant^1^, increased brain size in the fetus^2^, and constrains of the bipedalism and straight posture on the size and shape of the maternal pelvis^3^ in humans are associated with numerous potential opportunities for complications^4^.

The Old World non-human primates (NHP) - baboons (*Papio spp*.)^5^ share similarities with humans regarding mechanisms, regulating reproduction^6^, fetal and placental development and some pregnancy-related complications^5,7,8^. However information about the mechanism of birth and the complications arising during parturition in these species is relatively sparse.

The observations by Yeligulashvili^9^, representing cumulative data on over 200 births spanning a 16 year period in the Sukhumi nursery, remain one of the largest reports documenting labor in NHP. The author catalogued a number of observations on conditions associated with reproductive success in this colony and reported several characteristics of baboon parturition including the relative frequency of fetal presentation in labor (most often a face presentation with the head fully extended) and that the vast majority of births happened at night. He reported that delivery in the breech position was associated with a high rate of loss, recalling only two live births delivered breech. The nocturnal predilection of time of birth was thought to allow the pregnant female to move with the troupe during daytime to forage and not to be caught alone and relatively unprotected due to a daytime delivery. The nocturnal switch in the frequency and amplitude of the uterine contractility has been confirmed in these species by Nathanielsz’s group^10^. Although the uterine contractility patterns around the time of deliveries have been described in detail^11,12^ direct observations of the birthing process have only rarely been reported^9,13–15^.

In this manuscript, we add information from a series of pathological and observational cases to highlight insights and selected complications of birth in *Papio spp*. based on video-recording of the delivery process, X-ray, MRI and ultrasound evaluations in pregnant baboons, and pathology records obtained from perinatal loss in a large baboon colony during a 17 year period^5,16^.

## Materials and Methods

### Animal housing, characteristics and study design

Animals were housed in animal facilities at the Southwest National Primate Research Center, Texas Biomedical Research Institute (SNPRC), as described in detail elsewhere^5^ and in individual cages in the ALLAAC approved animal facilities at the Department of Comparative Medicine, University of Tennessee Health Science Center^17^. All protocols were approved by Texas Biomedical Research Institute, University of Texas at San Antonio, or University of Tennessee Health Science Center IACUC Committees and all methods were performed in accordance with the relevant guidelines and regulations. The detailed pathological evaluations were performed in cases of maternal and fetal death/euthanasia. Pathology records (17 years) of the baboon colony were analyzed for the presence of dystocia and selected cases with documented diagnosis of dystocia are reviewed in the present manuscript. Additionally incidental findings pertaining to pregnancy and dissection of the pelvic floor in diseased animals were performed. The characteristics of the animals are listed in Table 1.

**Table 1:** Characteristics of the animals and procedures described in the study.

Case number	Fetal weight (g)*		Fetal sex		Gestational age (dGA, days of gestation)			Gross description (if available)	Procedures performed		Clinical presentation and diagnosis
**Dystocia: face presentation**											
1	1068		male		term			Massive oedema	Routine necropsy		Dystocia, stillbirth
2	1248		male		179.6			N/A	Emergency C-section, routine necropsy, bacteria in placenta		Dystocia, stillbirth
3	764		female		Not reported			The face had area of hemorrhage, especially on the muzzle	Routine necropsy		Probable asphyxia, dystocia, stillbirth
4	784		male		Not reported			The face was reddened over the muzzle	Routine necropsy		Dystocia, stillbirth
5	924		male		187			N/A	Emergency C-section, routine necropsy		Painful, prolong contractions, dystocia, stillbirth
6	850		male		177			N/A	Routine necropsy		Dystocia, stillbirth
7	1106		male		191			N/A	Routine necropsy		Birth-trauma, probable dystocia, live birth, subsequent death
8	909		female		179			N/A	Maternal necropsy, photographs		Uterine rupture, maternal death
**Dystocia: Breech presentation**											
9	1146.1		male		180			N/A	Emergency C-section, routine necropsy		Breech presentation, stillbirth
10	811.6		male		Not reported			N/A	Routine necropsy		Probable trauma at birth due to breech presentation
**Maternal complications**											
***Case number***	***Maternal age*** **(** ***years*** **)**			***Gestational age (dGA)***			***Clinical Information***			***Procedures performed***	
11	18			Appr.150			Geriatric, weak, humane euthanasia			Morphometry of uterus, fetus	
12	15			192			Large Trichobezoar in the stomach, maternal death			Morphometry of uterus, fetus	
13	8			At term			Retained placenta, severe hemorrhage, humane euthanasia			Necropsy, examination of uterus and placenta,	
14	8			N/A			Histoplasmosis, humane euthanasia			Pelvic floor dissection	
15	N/A			N/A			N/A			Pelvic floor dissection	
**Observations of non-complicated pregnancies/deliveries**											
***Case number***		***Maternal age*** **(*years***)				***Gestational age*** **(** ***dGA*** **)**			***Procedures performed***		
16		N/A				165			Cesarean section, photographs, oligo-hydramnion		
17		17				147			Fetal MRI		
18		9				163			Ultrasound evaluation prior to delivery		
19		N/A				163			Ultrasound evaluation		
20		11				182			Video recording of delivery		

(N/A designates that information did not apply to a particular entry or not available); *the reported weight of healthy newborn baboons at the SNPRC colony is 888 g (min 480 g and max 1200 g)^39^.

## Results

### Dystocia cases (#1- #10)

Diagnosis of labor dystocia in the pathology records was documented in 70 cases, including 15 cases of breech presentation during the 17 years’ time period. During this period, there were 1021 cesarean deliveries, 5195 vaginal deliveries, 191 stillbirths/abortions with total number of deliveries 6449 and 1.09 cases of dystocia per 100 deliveries.

Photographic documentations of the fetal and or maternal condition were available in ten of the 70 cases (Table 1). All but one of the photo-documented cases, were associated with facial presentation and fetal demise. Additional morphometric fetal measurements were available for each case of dystocia and are presented on Table 1S (Supplementary material). In two out of ten cases the fetus was female. Fetal weight ranged from 764 g to 1248 g and the weight of the fetal brain from 80.4 g to 107.3 g.

**Figure 1:**
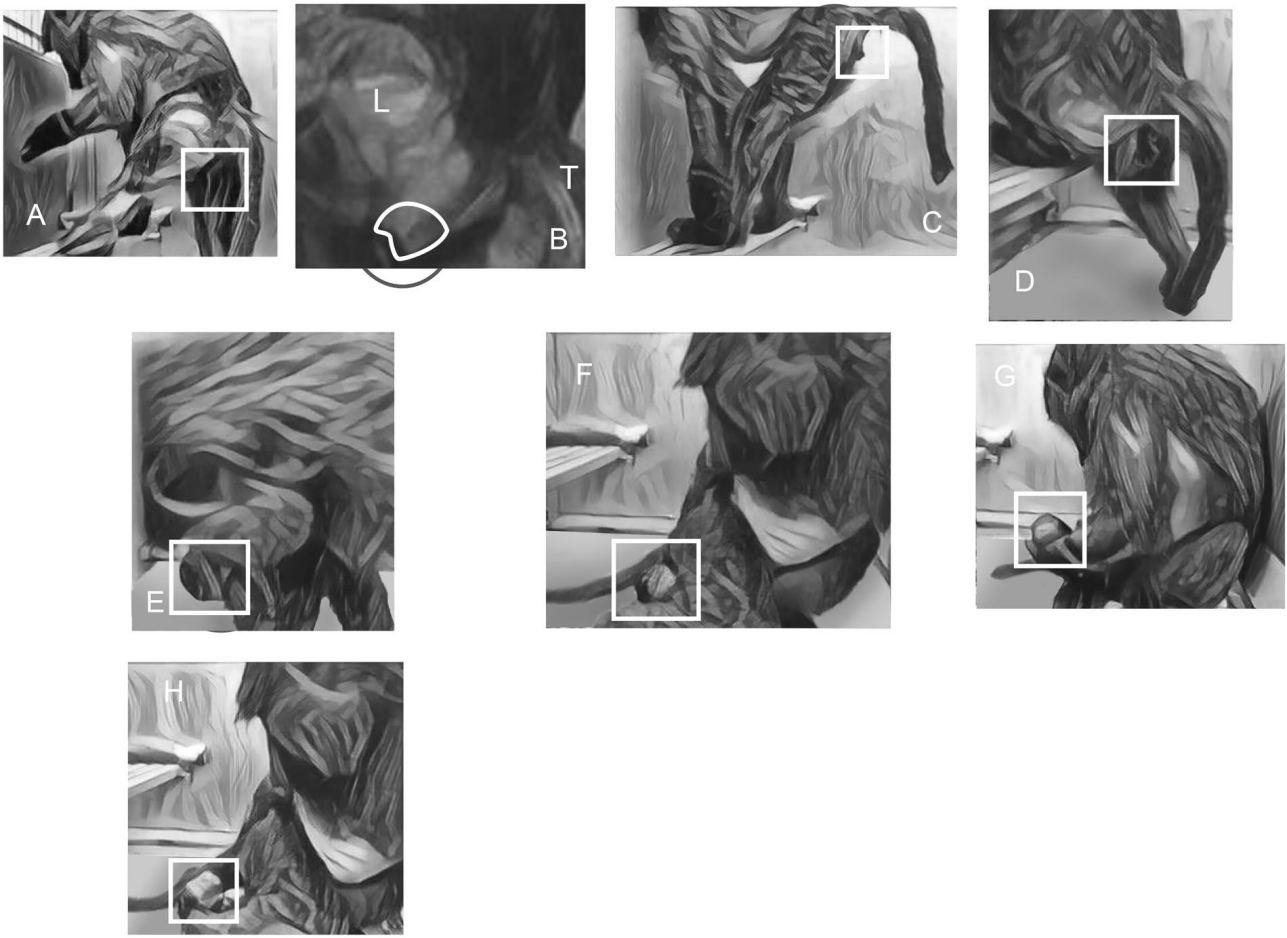
(**A**) initial brief pushing efforts, (**B**) the snout is visible through the introitus, (**C**) the presented part of the fetus did not stay on the perineum at the introitus between expulsive efforts, (**D**) snout fixation, (**E**–**G**) External head rotations, (**H**) fetus is born in prone anterior position.

The gross pathological appearance of fetuses of dystocia cases had one common feature - noticeable edema and bruising of the snout (Figure 1S.1A, case #2, Supplementary material) with bruising in frontal and midsagittal areas of the skull and absence of scalp molding (Figure 1S.1B, Supplementary material). In cases #2 and #5 there was visible bruising at the low uterine segment and utero-cervical junction suggesting that there had been a protracted dystocia (Figure 1S.1C and D, case #2, Supplementary material). In case #8, a posterior rupture of the cervix/vaginal junction took place (Fig. 1S.2, Supplementary material). In all three these cases (#2, #5, and #8), the fetal faces were engaged in a partially dilated (2.5–3 cm) cervix or pelvis.

### Maternal complications

#### Cases #8 and #13

Maternal delivery complications included cases of uterine rupture and retained placenta. Maternal humane euthanasia due to deteriorating maternal conditions was performed in severe cases. The examination of the uterus in case #13 showed a short, dilated cervix, and lower uterine segment (Figure 1S.3A,E, Supplementary material), placenta was adherent in the uterine fundus (Figure 1S.1B, C and D, Supplementary material).

#### Cases #11, #12 and, #16

At 147 days of gestation (human equivalent of 32 weeks gestation) (Fig. 1S.4A and B), case #11) the fetus had occipital presentation and the cervical canal was closed. The same finding was present in case #12 and #16 (Fig. 1S 4, Supplementary material).

### Uncomplicated deliveries

#### Imaging (case #17)

***MRI****.* The MRI picture, obtained at 147 days show that the uterine wall thickness in the baboon is less than that seen in the human uterus when analyzed at a comparable gestational age. There is also less fetal adipose tissue and a lesser amount of amniotic fluid around the fetal baboon compared to that typically seen around the human fetus at an equivalent gestational age (Figure 2S, Supplementary material).

***Ultrasound evaluation***. (cases #18 and #19). The ultrasound evaluation of a term baboon fetus (Figure 3S, Supplementary material) shows the snout engaged in the pelvis shortly prior to the onset of labor (Figure 3SA, Supplementary material). The head was extended with facial presentation. The length of the cervix was 4.02 cm (Figure 3SB, Supplementary material). These images were obtained during performance of an experimental protocol^17^. The fetus was externally rotated into the breech presentation for the experimental intervention and then positioned back into cephalic presentation at the end of the surgery. Pre-intervention ultrasonography revealed low amniotic fluid volume; therefore 250 ml of warm saline solution were instilled during the procedure to facilitate the manipulation and operative intervention. It was observed that this infusion resulted in dilation of internal cervical os and eventual rupture of the fetal membranes. The animal delivered a healthy female infant 72 h after the fetal procedure.

***Video-taping*** **(*case #20*)**. We were able to capture the birth process of a baboon Case #20 on videotape (Addendum). During the second stage of this videotaped labor, several maternal expulsive bursts were observed in rapid order of initial brief (5 seconds) pushing efforts brought the snout down to be visible through the introitus, but the presented part of the fetus did not stay on the perineum at the introitus between expulsive efforts (Fig. 1A–C). After three sucessive efforts, a sustained push with a duration of 17 second resulted in snout fixation (Fig. 1D). During the pentultimate pushing effort which lasted eight seconds, the head was born in a facial anterior presentation (Fig. 1E–G). Following expulsion of the head pointed toward the ventral side of the mother, it rotated 90° and a final push effected delivery of the body of the newborn so that the fetus was able to placed on the ground in prone anterior position. Placenta was delivered shortly after and eaten. The animal did not vocalize during labor.

#### Anatomy of the pelvic floor (cases #14 and #15)

Pelvic dissections in old nulliparous (Figure 4SA, Supplementary material) and young multiparous (Figure 4SB, Supplementary material) baboons showed, that vaginal musculature was enveloped in well-developed connective tissue which was dissected from the levator complex (Figure 4SA,B Supplementary material). The *pubocaudalis* extended from the medial surface of the puboischiatic symphysis to converge and attach to second and third caudal vertebrae. The *iliocaudalis* originated directly from the pelvic brim. The *arcus tendineus* fascia pelvis were absent in baboons. The vaginal muscularis was enveloped in connective tissue that suspends the vaginal wall and urethra to the pubo- and *ilio-caudalis* musculature. The homologous puborectalis muscles were absent; the *retractor recti* originated at the sacrum and encircled the anterolateral sides of the rectum.

## Discussion

Observations of labor and deliveries in baboons and macaques (rhesus and lapunders) were described by Yeligulashvili in 1955^9^ in the largest-to-date study regarding documentation of labor in non-human primates (106 baboons and 117 macaques). The author observed that the majority of pregnant dams are carrying a fetus in a longitudinal position during the last month of pregnancy and frequency of delivery in facial presentation was 62% and occipital presentation was 37% among fetuses delivering in the vertex presentation. This high rate of extended face presentation stands in contradistinction to chimpanzees and humans which have almost exclusively occipital presentation during labor. Birth in chimpanzees occurs predominantly in the posterior occipital position, while in humans this position is present in only 3% of deliveries^12,18^. Stoller performed radiographic evaluation of the delivery process in four baboons^19^ and described head extension and facial presentation in all cases studied. He also reported a breech delivery with an intrapartum death. In our case series, we showed the presence of head extension and the beginning of cervical dilation on ultrasound examination at least 2 days prior to delivery.

Because of the high incidence of reproductive loss, some veterinarians have recommended external version of the baboon fetus in case of breech presentation as an alternative to either vaginal breech or Cesarean delivery of the fetus in labor^20^. It is difficult to estimate the number of perinatal losses due to breech presentation in baboons but historical and anecdotal data from veterinarian experiences support the fact that it is a prominent reproductive hazard. However, the baboon fetus has higher mobility, compared to human: Yeligulashvili described numerous instances of the fetus in a breech presentation within 24 h of delivery, only to spontaneously convert to a cephalic presentation for birth. He reported about a 16% rate of breech delivery and a large stillbirth risk associated with this delivery position, recalling only two live births from a breech fetus even with assistance at delivery.

The reasons for the poor survival with breech birth may be due to a combination of factors. In our work, we show that the hip circumference in the fetal baboon is less than the abdominal, chest and head circumferences. Since the breech presentation is a compound one, (with feet and buttock as presenting parts), the total circumference of the dilating wedge could be comparable with the head circumference, but more likely the dilating wedge is smaller. Partial delivery through an incompletely dilated cervix may lengthen the transit time with a breech presentation beyond the usually short birth process. In addition, in contrast to the head extension seen in most baboon deliveries, a breech delivery leads to a prolonged delay in the clearance of the airway at delivery increasing the likelihood of birth asphyxia.

The decreased amount of amniotic fluid combined with the thinner uterine wall at the end of gestation, and absence of the well-developed lower uterine segment, compared to humans, might be involved in the mechanism of the head extension rather than flexion (observed in *Homo sapiens*) of baboon fetuses prior to their delivery. Based on these data the dimensions of the fetal head to be measured for the accessing labor prognosis, delivery and fetal development in the baboons should be *submento-bregmaticus*, *sub-occipito-bregmaticus* and *verticomento* diameters^19^.

The baboon fetus has less percentage of adipose tissue and a greater degree of muscle tissue compared to human fetus^21^. This difference might affect timing of labor, since subcutaneous adipose tissue may function as an endocrine organ which interacts with the placenta^22,23^.

We have previously reported on reproductive loss in the baboon^5,16^ and in the current report we have included a number of cases which highlight both the normal mechanisms of labor as well as traumatic causes of reproductive loss. The investigation into causes of loss in baboons is hampered by the fact that the birth is usually not witnessed and the majority of baboon placentas are consumed at birth and therefore not available for pathologic evaluation^5^.

It appears that while the mechanisms of birth in baboons are less complicated compared to humans, traumatic delivery and dystocia still are factors in the reproductive success^16^. The incidence of the dystocia, reported in this manuscript, is less than reported for some geographical regions in humans (12.2%)^24^, but within the range with some other reports (0.96%)^25^. The fetal baboon head does not undergo much molding during labor^19^ and the complex rotational movement that the human fetus experiences to exploit the gynecoid birthing adaptations of the female human pelvis are not seen in NHP^26,27^. The baboon parturient delivers her fetus without assistance^13,15^. Breech delivery appears to be a traumatic event in both the human and NHP^28^. Similarly, absolute cephalopelvic disproportion and birth dystocia can lead to both maternal and fetal morbidity and mortality^29,30^.

Duration of the third stage of labor which is the time from delivery of the fetus to expulsion of the placenta has been reported to last up to two hours in baboons in the wild and pathologic retained placenta is rare in these species^31^. However, in the baboon breeding colonies retained placenta was the most common cause of clinical admission^32^ and severe bleeding, leading to maternal death/euthanasia was documented in 0.6% cases^32^. Despite this fact, the diagnosis *of placenta accreta* is non-existing in the primatology bibliography. Documented in the present study case is the only one, indicating possible presence of abnormally deep invasion in this species, which parallels cesarean scar placentation in humans^33–35^.

We were not able to observe clear separation of the puborectalis part of the levator ani muscle groups in the baboon, while other authors described such presence^36,37^. Interestingly, vaginal prolapse (associated the damage of the *puborectal* muscle in humans) is very rare in baboons^38^.

In conclusion, the presented cases provide examples of normal and pathologic instances of the parturition process in the baboon. From these data, important information for the management of pregnancy and delivery in the non-human primates can be inferred.

## Electronic supplementary material

Supplementary table and figures

Delivery Video
