# Supplementary material for: Parturition in baboons (PAPIO SPP.)
Source: Sci Rep. 2018 Jan 19;8:1174. doi: 10.1038/s41598-018-19221-4 (PMC5775344; doi:10.1038/s41598-018-19221-4)

PARTURITION IN BABOONS (*PAPIO SPP.*)

Short Title: PARTURITION IN BABOONS

SUPPLEMENTRARY TABLE AND FIGRUES

N. Schlabritz-Loutsevitch[1*], J. Maher[1], R. Sullivan[2], G. Mari[3], M. Schenone[3], H.L. Cohen[4], R.A. Word[5], G.B. Hubbard[6], E.J. Dick Jr.[7]

[1]Department of Obstetrics and Gynecology, College of Medicine, Texas Tech University Health Sciences Center at the Permian Basin, Odessa, TX, USA

[2] Department of Comparative Medicine, University of Tennessee Health Science Center, Memphis, TN, USA

[3] Department of Obstetrics and Gynecology, University of Tennessee Health Science Center, Memphis, TN, USA

[4] Department of Radiology, University of Tennessee Health Science Center, Memphis, TN, USA

[5]University of Texas Health Sciences Center at Dallas, Dallas, TX, USA

[6]University of Texas Health Sciences Center at San Antonio, San Antonio, TX, USA

[7]Southwest National Primate Research Center, Texas Biomedical Research Institute, San Antonio, TX, USA

**Corresponding author*
Natalia Schlabritz-Loutsevitch[1]
Associate Regional Dean for Research
Associate Professor (Research)
Department of Obstetrics and Gynecology
Department of Pharmacology and Neuroscience
Texas Tech University HSC School of Medicine
e-mail: Natalia.schlabritz-lutsevich@ttuhsc.edu
office phone: 432-703-5169
cell phone: 210-317-0156
http://www.ttuhsc.edu/odessa/research/schlabritz_lab.aspx
https://www.facebook.com/Texas-Tech-University-Health-Science-Center-in-the-Permian-Basin-Research-1612649318999433/

Table 1S. Fetal morphometries of ten cases of dystocia.

Case Number / Organs	1	2	3	4	5	6	7	8	9	10
Weight (g)	1068	1248	764	784	924	850	1106	909	1146.1	811.6
Fetal sex	M	M	F	M	M	M	M	F	M	M
Length (cm)	43	46	37	42	40		44.5	40	43.5	37.5
Fronto-occiptal distance (cm)		13		12	14.5		10.5			
Biparietal distance (cm)				6.2	5.8		6.1	6.2	6.5	7
Head circumference (cm)		24	21	22	23.5		25.2		23.5	
Abdominal Distance (cm)	8.5	9.5	8	3.3	4.5		6.1	5.8	3	6.5
Manubrium-pubic distance (cm)	14	15.5	15.5	16	14		15.5	13	16	14.5
Femur length (cm)	8	7.5	8	8	8		8.5	7.5		7.5
Weight placenta (g)		328			151.5		102.1	150	240.6	109.2
Length of umbilical cord (cm)		25	33		22			26	42	20
Length of stomach (cm)		5	2.5	5	3.3		5			4.5
Anus to ileocecal junction (cm)		21.5	41.5	63	45		55.5			44.5
Ileocecal junction to pylorus (cm)		31	61	89	100		73.5			62.5
Chest circumference (cm)	20.5	20	15	16			20	18	19	17.5
Waist circumference (level of umbilicus) (cm)	15.5	18	14	15	13.5		17	14	16.5	16.5
Hip circumference (cm)	15.5	20	15	15	13.5		17.5	18	20	14.5
Brain weight (g)	80.4	94	85.1		90.4		103.9		107.3	
L. Kidney weight (g)		2		2.95	3.4		2.9		3.6	
R. Kidney weight (g)	2.6	2	6	2.78	3.27		2.7			
Liver weight (g)	46.1	60	48	34	23.3		35.9			
L. adrenal weight (g)		0.435		0.397	0.38		0.589			
R. adrenal weight (g)		0.238	1	0.282	0.32		0.519			
Spleen weight (g)	138	2.3	2	2.21	1.5		1.5		1.6	
Heart weight (g)	7.0	4	6	4	5.6		8		7.7	
Thyroid weight (g)	0.398	0.705	0.416							
Lung weight (g)	19.0	20	10	16	9.4		16.4		21.3	

Figure 1S. 1. Protracted labor. Stillborn fetus at term. (1A) edema and bruising of the snout (1B) hemorrhages in frontal and midsagittal areas and absence of the scalp molding in the same fetus. Uterus after protracted labor. (1C) hemorrhages at low uterine segment (blue arrow) (1D) inner surface (blue arrow) of uterine-cervical junction in the same animal. **2.** Uterine rupture (case #8) (2A) engagement of fetal head into maternal pelvis, (2B) posterior rupture of uterus, (2C) anterior surface of ruptured uterus. Note: full urinary bladder. **3.** Hysterectomy specimen of animal with the diagnosis of retained placenta (3A) anterior view of 1. Cervix, 2. Lower segment, & 3. *Corpus uteri*. (3B) longitudinal section of uterus. (C) and (3D) area of *placenta accreta* in the area of cesarean section scar, and (3E) dilated cervix (5 cm). **4.** (4A) Pregnant uterus at 147 days of gestation and (4B) radiography of fetal position, (4C) pregnant uterus at the end of gestation and (4D) fetal position, (4E) pregnant uterus at term (cesarean section), Fetal growth restriction and olgohydramnion. P-placenta, U- uterus, asterisk fetal head, arrowhead – presenting part.

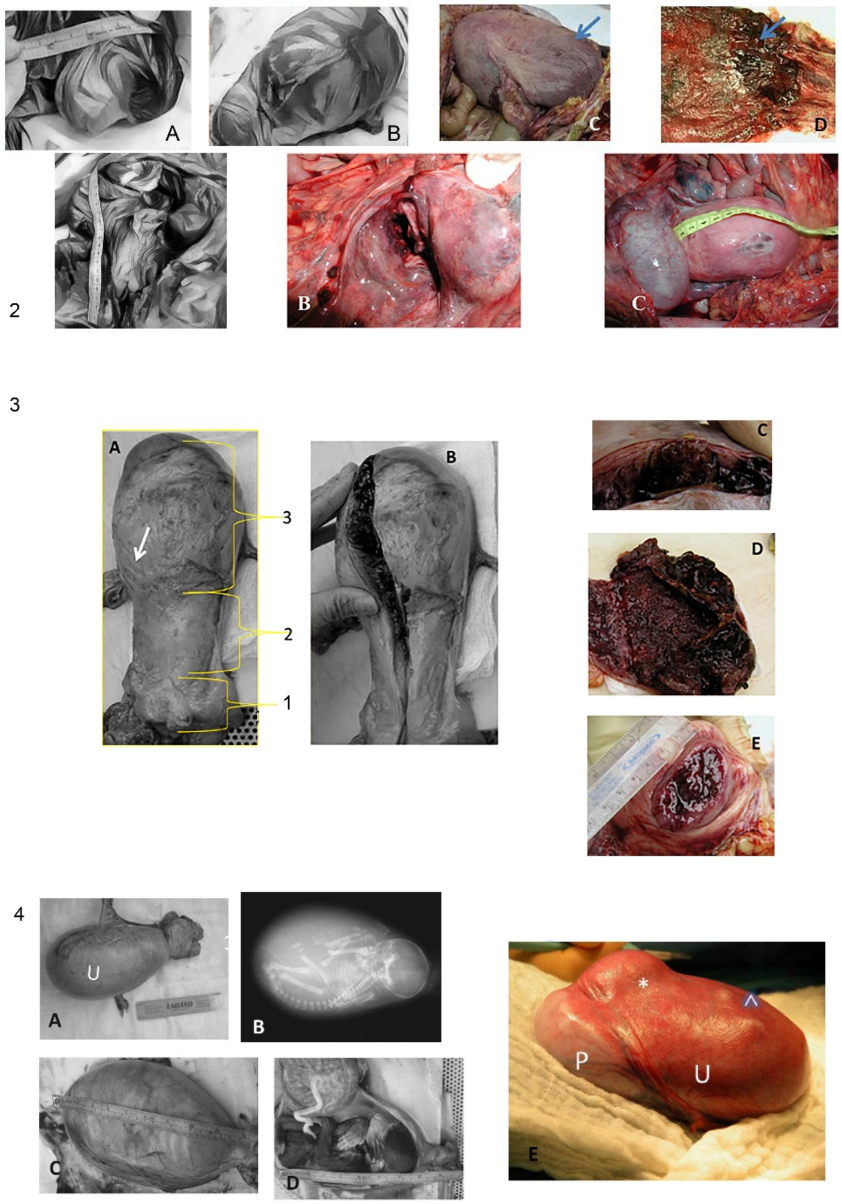

Figure 2S. MRI images of baboon (A) and human fetuses (B) in breach presentation, similar gestational age. Note: absence of subcutaneous fat in the baboon fetus.

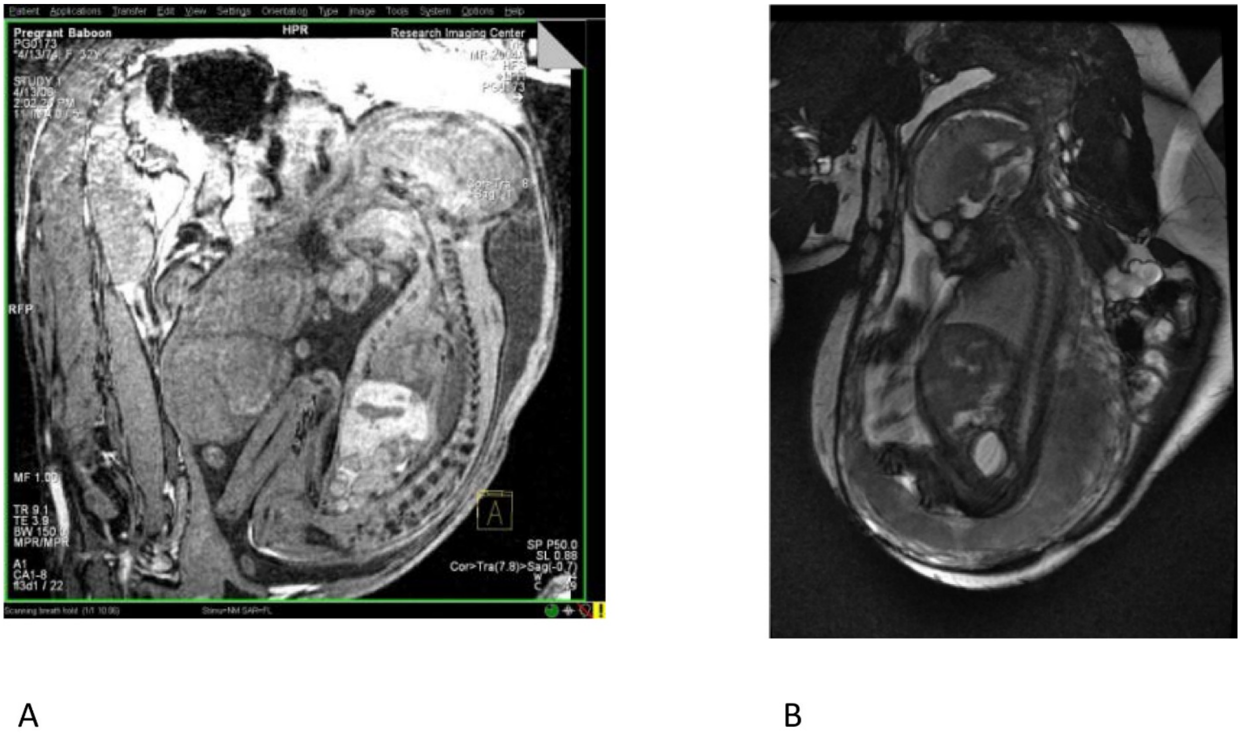

Figure 3S. Ultrasound image of (A) fetal baboon at term with snout (arrow) engaged into the cervix, (B) partially dilated cervix (same animal).

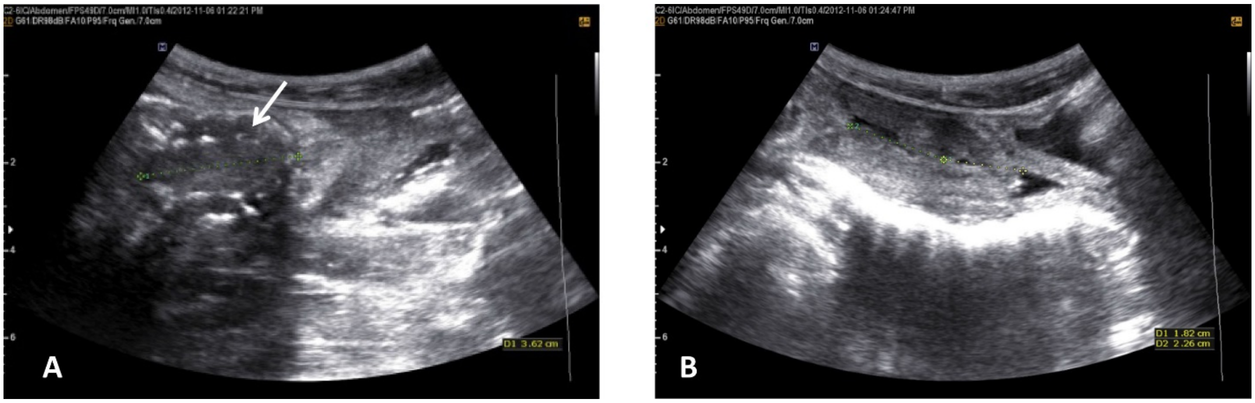

Figure 4S. (A) Dissection of space of Retzius and identification of levator ani: PC – *m. Pubocaudalis Iliocaudalis* originates directly from the pelvic brim rather than the *arcus tendineus fascia pelvis* (absent in baboons). (B) *Levator ani*.

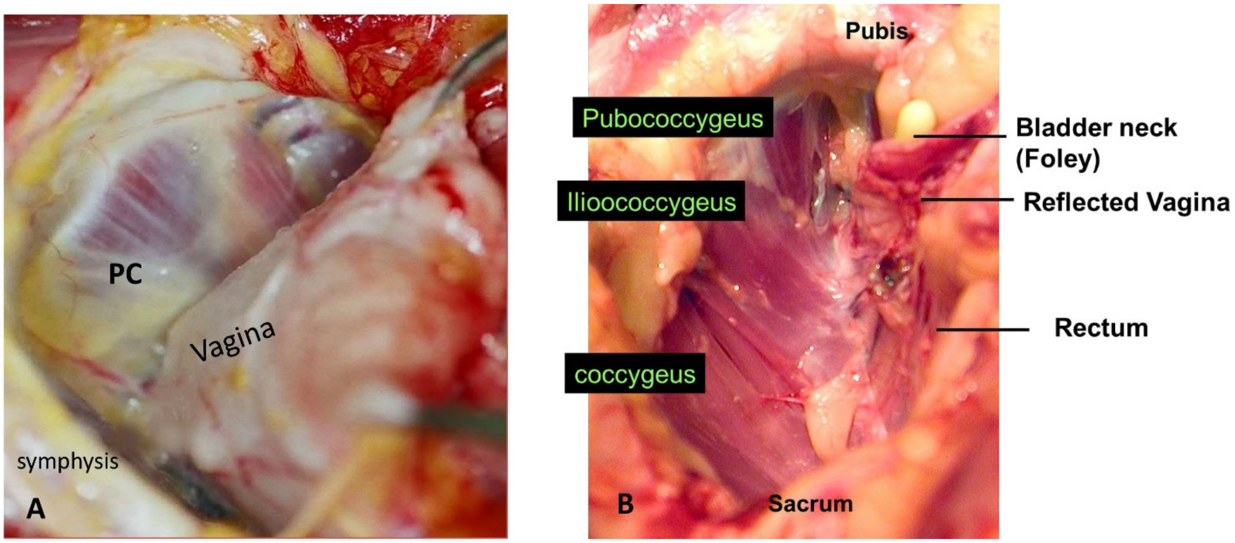

Supplement: Supplementary file 1 — Supplementary table and figures [file 41598_2018_19221_MOESM1_ESM.pdf]
