# Supplementary figures and images for: Parturition in baboons (PAPIO SPP.)
Source: Sci Rep. 2018 Jan 19;8:1174. doi: 10.1038/s41598-018-19221-4 (PMC5775344; doi:10.1038/s41598-018-19221-4)

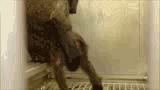

Supplement: Supplementary file 2 — Delivery Video [file 41598_2018_19221_MOESM2_ESM.gif]
